# Effects of in ovo acrylamide injection on bone mineral composition and morphometric, geometric and mechanical parameters of femur and tibia bone of newly hatched chickens

**DOI:** 10.1016/j.psj.2026.107816

**Published:** 2026-09-21

**Authors:** J. Wojciechowska-Puchałka, J. Wieczorek, K. Kustra, M. Trela, S. Muszyński, E. Tomaszewska, M.W. Lis

**Affiliations:** a Department of Animal Reproduction, Anatomy and Genomics, University of Agriculture in Krakow, Mickiewicza Alley 24/28, Krakow, 30-059, Poland; b Department of Agricultural and Environmental Chemistry, University of Agriculture in Krakow, Mickiewicza Alley 21, Krakow, 31-120, Poland; c Department of Zoology and Animal Welfare, University of Agriculture in Krakow, Mickiewicza Alley 24/28, Krakow, 30-059, Poland; d Department of Biophysics, University of Life Sciences in Lublin, Akademicka 13, Lublin, 20-950, Poland; e Department of Animal Physiology, University of Life Sciences in Lublin, Akademicka 12, Lublin, 20-950, Poland

**Keywords:** Acrylamide, Biomechanics, Chick, Long bones, Skeletal changes

## Abstract

Acrylamide (**ACR**) is a recognized toxic compound that can contaminate food and animal feed and thus pose a risk of transfer to eggs. Due to the fact that this substance has a negative effect on the neural tube. We decided to test the hypothesis that ACR leads to weakening bone strength in long bones of chicken chicks and, consequently, to motor coordination disorders. Therefore, the aim of this study was to determine the effect of administration of ACR *in ovo* at various doses (0.25 mg, 0.5 mg, and 1 mg/egg) on the mechanical, morphometric and geometric properties of the tibia and femur, as well as indicators of mineralization/bone formation in the body (Ca, P, Ca:P, ALP) among one-day-old chicks. Chicken embryos on day 6 of incubation were divided into 6 equal experimental groups (n = 96 per group), which were injected with the following substances: (1) 0.9% saline solution (0-ACR), (2) ACR at a dose of 0.25 mg/egg (L-ACR), (3) ACR at a dose of 0.5 mg/egg (M-ACR), (4) ACR at a dose of 1 mg/egg (H-ACR), (5) ACR at a dose of 2 mg/egg and (6) ACR at a dose of 4 mg/egg. Hatchability was 74.0, 67.7, 66.7 and 49.5% in groups 0-ACR, L-ACR, M-ACR and H-ACR, respectively, whereas in the groups treated with doses of 2.0 and 4.0 mg ACR per egg, all embryos died. Statistical analysis revealed a varied and dose-dependent effect of ACR on the geometric, osteometric, and mechanical properties and mineral composition of the long bones of one-day-old chicks. The administration of ACR at a dose of 0.5 mg/egg resulted in an increase in the vertical external diameter (**VED**) (P = 0.025) and radius of gyration (**R_g_**) (P = 0.034) in the tibia, the vertical internal diameter (**VID**) (P = 0.014) in the femur, as well as the calcium (**Ca**) (P = 0.020) and phosphorus (**P**) (P = 0.041) content in pooled femur and tibia samples relative to the control group. In addition, an increase in ultimate load (**F_max_**) (P = 0.022) and work to fracture (**W_max_**) (P = 0.036) was demonstrated, but only in the tibia in the group treated with ACR at a dose of 0.25 mg/egg compared to the group injected with 0.9% saline solution. Furthermore, elevated strains (**ε_y_** and **ε_max_**) of the tibia were observed at all applied doses (0.25, 0.5, and 1 mg/egg) (P = 0.002, P = 0.003, P < 0.001 for **ε_y_**, respectively, and P = 0.009, P = 0.010, P = 0.016 for **ε_max_**, respectively) and higher ultimate strain (**ε_max_**) (P = 0.032) of the femur in the M-ACR group. However, the body weight of birds as well as Ca, P, Ca:P, and alkaline phosphatase (**ALP**) levels in blood remained at similar levels in all experimental groups. In summary, the findings do not indicate generalized weakening of long bones at hatch. Instead, they show endpoint- and bone-specific responses whose biological persistence and mechanism remain to be established. Thus, these studies advance understanding of long bone development and bone mineralization among one-day-old chicks following ACR administration and provide a basis for continuing research beyond the hatching period to determine the likelihood of health effects later in life.

## Introduction

Food is one of the basic factors determining the proper functioning of the body and is essential for life. Therefore, providing food with the right energy value ensures proper growth and development of the body. Unfortunately, current mass food production is associated with low-quality and highly processed products (Monteiro et al., 2018; Prache et al., 2022). It is widely known that, in addition to nutrients, other substances can enter the body that have a negative impact on its functioning (Aljohani, 2023; Chen et al., 2018). One such compound commonly observed in the human and animal environment is ACR. This toxic material can be formed during the heat treatment of food products (e.g. during the Maillard reaction) and can also be synthesised on an industrial scale (Bent et al., 2012; Kepekci Tekkeli et al., 2012; Stadler et al., 2002).

Several studies indicate the presence of ACR in many food products, i.e. chips, fried potatoes, tea, instant coffee, breakfast cereals, as well as in livestock feed and, consequently, in milk and eggs (Becalski et al., 2003; Kienzle et al., 2005; Kumar et al., 2018; Pabst et al., 2005). In the case of poultry, only Halle et al. (2006) conducted an experiment in which female Lohmann select Leghorn hens were exposed to ACR administered in feed at a dose of 671 ± 32 µg per kg, which resulted in the substance transferring into the egg at a maximum concentration of 17.2 µg/kg of egg weight (and the carry-over rate from feed to eggs was 0.022 for hens in the experimental group and 0.010 for hens in the control group.) Based on this, it can be assumed that there is a risk of exposure of chicken embryos to ACR. However, the authors did not conduct analyses of fertilized eggs, which constitutes a significant research gap. Nevertheless, it is known on this basis that the concentration of ACR in starch-based poultry feed may exceed 0.5 mg/kg. As indicated by Batoryna et al. (2018), such concentrations in animal feed may correspond to the doses used in toxicological studies on chicken embryos, which is important from a biological perspective. Moreover, it is used in industry and agriculture in its polymerised form - polyacrylamide. Polyacrylamides are massively used in the production of adhesives, varnishes, paints, fertilisers or plastics (Elias et al., 2017; Stošić et al., 2018). In addition, polyacrylamide gels are used in molecular biology for protein and DNA electrophoresis. However, polyacrylamide is chemically stable and non-toxic, and the risk of its exposure concerns only the trace amounts of ACR monomers. Therefore, exposure to this toxin represents not only food but possibly also with environmental contamination. Numerous studies confirm its presence in surface water, drinking water, and soil (Backe et al., 2014; Oh et al., 2026). Thus, Naiel et al. (2023) confirm the presence of ACR in river water and tap water at levels below 5 µg/L, and in drinking water at levels ranging from 0.1 to 500 µg/L, which can cause contamination of both water and soil. Consequently, there is a risk that poultry flocks may be exposed to this compound not only through feed but also through water.

ACR can penetrate the organism through the oral, inhalation, skin, blood/placenta and blood/milk ways, (Fuhr et al., 2006; Mahmoud et al., 2023). Batoryna et al. (2017, 2018) using an *in ovo* chick embryo model, found that ACR exerts a significant influence on the redox balance in the developing brain by impacting the activity of antioxidant enzymes and the levels of glutathione (**GSH**). Thus, it is an agent highly genotoxic, hepatotoxic, nephrotoxic, neurotoxic, mutagenic, toxic to development and reproduction in animals (Gupta et al., 2023; Zhang et al., 2013). However, its neurotoxic effects and potential carcinogenicity have been well documented among people (Govindaraju et al., 2024). That is why many global organizations, such as the World Health Organization (**WHO**), Food and Agricultural Organization (**FAO**), International Agency for Research on Cancer (**IARC**) have recognized ACR as a substance hazardous to health and life (Başaran et al., 2023; Sarion et al., 2021).

The chicken embryo is a valuable *in vivo* model and an ethical alternative to mammals, in accordance with the principles of reduction, replacement, and refinement (the **3Rs**). This concept aims to maximize the benefits of an experiment for humans, other organisms, and the environment, while causing as little suffering as possible to the fewest number of animals. The 3R rule regulates the conduct of necessary animal experiments while ensuring the animals' welfare and maintaining public acceptance (MacArthur Clark, 2018). According to this principle, the use of avian embryos makes it possible to minimize pain and stress, as well as to reduce the number of other animal species used as *in vivo* models, by creating an alternative that does not pose significant technical or ethical challenges (Zosen et al., 2021). *In ovo* injection involves inserting a needle through the eggshell to administer a substance directly into the egg, most often into the air chamber or directly into the embryo itself (El-Sabrout et al., 2019). Therefore, *in ovo* models also serve as a very good alternative to *in vitro* studies, which are subject to limitations and errors compared to *in vivo* systems. As a result, chicken embryos are used in toxicological, oncological, and pharmacological research, in poultry vaccines, and in the study of embryonic development (Augustine et al., 2019; Ghimire et al., 2022; Kaplan-Arabaci et al., 2025). This is possible since laying hens (especially those of commercial breeds) provide a large number of embryos with similar genetic backgrounds and stable phenotypes (Gong et al., 2023). Due to these characteristics, chicken embryos serve as a valuable model characterized by high reproducibility, autonomous development, uniform size, and ease of manipulation (Kaplan-Arabaci et al., 2025). Consequently, it serves as a good comparative model for other vertebrates, mainly in terms of embryonic development, which makes chicken embryos a very attractive research model (Rashidi and Sottile, 2009) explains why they are successfully used in skeletal system research (Nowlan et al., 2010; Torres and Korver, 2018).

It is also worth noting that studies conducted on humans and animals (including chicken embryos) indicate that ACR has toxic effects on the neural tube, which can lead to developmental disorders (Becit-Kizilkaya et al., 2024; Lauvås et al., 2022). As a result, animals exposed to ACR may experience impaired coordination, limb weakness and eventually even paralysis. This is particularly important given that skeletal disorders in poultry are considered one of the most significant factors affecting the final outcomes of poultry production. In addition, significant increases of muscle mass (especially in the pectoral muscles) in broiler chickens and increases in the number of eggs by laying hens contribute to a higher incidence of diseases affecting this system and to stress, primarily on the pelvic bones (Alfonso-Carrillo et al., 2021; Fleming, 2008). Due to the rapid rate of growth and development, as well as the forward shift of the bird’s center of gravity, the tibia and femur are most susceptible to pathological changes. An imbalance between muscle mass and bone mass in broilers, as well as insufficient bone tissue growth in laying hens, leads to growth disorders, deformities, and fractures, and consequently to an increased incidence of metabolic bone diseases such as lameness, rickets, osteoporosis, osteomalacia, tibial twisting, tibial dyschondroplasia, and shaky leg syndrome (Cook, 2000; Kierończyk et al., 2017; Thorp, 1994). Furthermore, it has been documented that even in young chicks, shortly after hatching, leg splay, and curled toe paralysis can occur (Arya et al., 2025; Permana et al., 2023). Thus, exposure to ACR during the embryonic period may result in impaired musculoskeletal function in chicks immediately after hatching and throughout their subsequent lives. This can lead to problems with balance and mobility in these animals. It is worth noting that reduced mobility and a sedentary posture in birds often result not only in pain but also in a lack of interest in feeding and drinking, which in turn leads to deformities and muscle atrophy in the breast area and can cause infections and diseases (Božičković et al., 2026). Furthermore, this may lead to increased stress in the birds, resulting in a higher mortality rate (Kierończyk et al., 2017; Rokavec and Zupan Šemrov, 2020). Therefore, leg disorders in poultry lead to culling and are considered one of the causes of increased mortality, thereby resulting in significant economic losses (Szafraniec et al., 2022; Xu et al., 2025). It is estimated that in just the United States, financial losses resulting from skeletal disorders in the poultry industry range from 80 to 150 million dollars annually (Kierończyk et al., 2017; Sullivan, 1994; Szafraniec et al., 2022). Therefore, the issue of skeletal disorders in poultry is significant not only from the perspective of animal welfare, but also from an economic perspective.

It is important to note that the skeletal system’s ability to perform support functions depends on the mechanical properties of the bones, especially on their elasticity and strength to withstand loads acting in different directions and with different intensities. However, it should also be emphasised that the mechanical strength of bone depends on the mass of the bone, the microarchitecture, the geometric properties of the bone or the degree of mineralization (Ferretti et al., 1993). In a young organism, structural modeling predominates, while in a mature individual, the process of internal bone remodeling dominates (Seeman and Delmas, 2006). During structural modeling, the processes of osteogenesis prevail over those of osteolysis, resulting in bone growth in length and thickness, as well as an increase in bone mass. Toxic substances such as ACR can disrupt the balance between bone formation and resorption. This can consequently lead to abnormalities in bone microstructure, thereby weakening bone strength, which may result in serious bone diseases later in life. Therefore, the aim of our study was to evaluate the effect of exposing chicken embryos to ACR on the condition of bone tissue after hatching.

Although prenatal exposure to ACR impairs skeletal development in mammalian offspring, its *in ovo* effects on avian bone formation remain unexplored (Tomaszewska et al., 2020). We, therefore, investigated whether *in ovo* ACR, administered at various doses, alters development of the tibia and femur in one-day-old chicks, assessing whole-bone mechanical competence (three-point bending), bone geometry/osteometry, and systemic mineral/formation indices (plasma Ca, P, Ca:P, ALP). Given the tibia’s faster late-embryonic longitudinal growth, higher cartilage fraction at hatch, and its known susceptibility to limb disorders in poultry, we anticipated larger effect sizes in the tibia than the femur, manifesting as greater decrements in ultimate force and stiffness and less favorable geometric indices. Guided by these premises, we hypothesized that *in ovo* ACR exposure disrupts endochondral ossification and cortical modeling, leading to measurable weakening of long bones at hatch accompanied by adverse geometric changes and perturbations of mineral homeostasis.

## Materials and methods

### Ethical approval

The experiment was carried out in accordance with the instructions and with the approval of the 2nd Local Ethics Committee for Animal Experiments at the Institute of Pharmacology of the Polish Academy of Sciences in Cracow, Poland (No. 77/2025), European Union Directive No. 2010/63/EU and ARRIVE guidelines and all methods were performed in accordance with the relevant guidelines and regulations.

### Animal model and experimental groups

The experimental material consisted of hatching eggs (n = 600) weighing 60.2 g ± 3.32 g (mean ± SD) from a flock of Ross 308 broilers (Aviagen EPI in Zebowo, Poland; Gołaczewy Poultry Farm in Wolbrom, Poland). The eggs were set in the laboratory incubators (Iglotech, Cracow, Poland) and incubated at temperature 37.8 ± 0.1°C and relative humidity (**RH**) 50% ± 1% for 1st to 18th day of incubation and at temperature 37.2 ± 0.1°C and RH 55-70% for 19th–21st day of incubation. Until the 18th day of incubation, the eggs were placed on trays tilted at an angle of 45° and rotated by 90° every hour, while on the 19th day of incubation they were transferred to hatching baskets. All eggs were disinfected with ozone at a 0.5% working concentration prior to incubation using an BT-NT18 ozone generator (Bitom, Siemianowice Slaskie, Poland). On the 6th day of incubation, 576 fertilized eggs were selected, divided into six equally sized groups (n = 96) and administered the experimental substance in accordance with the recommendations of the Local Ethics Committee. Next, the eggshell surface on a big end of the egg was disinfected with 70% ethanol (Chempur, Piekary Slaskie, Poland), and a hole was made with an 18 G needle (1.20 mm × 40 mm). Then, 100 µl of solution was *in ovo* injected into the air chamber using a 1 ml insulin syringe with a G20 needle (0.9 mm x 40 mm) such a described Bojarski et al. (2022) and according to the experimental scheme presented below:

0-ACR – control group, injected with 0.9% saline solution (Polfa, Cracow, Poland)

L-ACR – I experimental group, injected with a solution containing acrylamide at dose of 0.25 mg per egg (A8887, Merck KGaA, Darmstadt, Germany)

M-ACR – II experimental group, injected with a solution containing acrylamide at dose of 0.5 mg per egg (A8887, Merck KGaA, Darmstadt, Germany)

H-ACR – III experimental group, injected with a solution containing acrylamide at dose of 1.0 mg per egg (A8887, Merck KGaA, Darmstadt, Germany)

IV experimental group, injected with a solution containing acrylamide at dose of 2.0 mg per egg (A8887, Merck KGaA, Darmstadt, Germany)

V experimental group, injected with a solution containing acrylamide at dose of 4.0 mg per egg (A8887, Merck KGaA, Darmstadt, Germany)

A solution of 0.9% saline provided the solvent for the ACR. The applied doses of the toxic substance, the volume of the solution and the time of application were the same as those described in our previous study (Wojciechowska-Puchałka et al., 2026) and were determined based on earlier research (Batoryna et al., 2018; Begum Sheikh and Kedam, 2010). After injection, the holes were sealed with hot paraffin and incubation continued (Bojarski et al., 2022; Tombarkiewicz et al., 2020). On the 10th and 19th days of incubation, the eggs were re-cancelled to remove dead embryos, and complete embryonic death was observed in the individuals from groups IV and V, which were treated with ACR at doses of 2 mg/egg and 4 mg/egg, respectively. All of the above experimental procedures were performed in the laboratory of Department of Zoology and Animal Welfare of the University of Agriculture in Krakow. After 21 days of incubation, hatchability was assessed, and 80 chicks were selected for further analysis (of which 40 were euthanized), while the remaining healthy chicks were available for adoption. All removed and unhatched eggs were broken and analyzed.

### Sampling

On the 1st day of life, 10 individuals were randomly selected from each group. Each animal was weighed (the body weight **BW** was determined) using the Adventurer Pro AV513CM electronic balance with an accuracy of 0.001 g (Ohaus Europe GmbH, Nanikon, Switzerland). Immediately after decapitation, blood (approximately 1 ml) was taken from the chicks' jugular vein into heparinized tubes (the procedures used to slaughter the animals were carried out in accordance with the applicable legal regulations: Council Regulation (EC) No 1099/2009 of 24 September 2009 on the protection of animals at the time of killing). Plasma was collected after centrifugation (3000 x g, 10 minutes, 4°C) and stored at −80°C for further analyses. The right tibia and femur were then carefully dissected and cleaned of soft tissue. Each bone was wrapped in gauze soaked in 0.9% saline solution, placed in a ziploc bag, frozen at −20°C and stored by the time morphometric, geometric and mechanical analyses were performed (Tomaszewska et al., 2024;Wojciechowska-Puchałka et al., 2023).

### Calcium, phosphorus and alkaline phosphatase determination in blood plasma

Plasma levels of ALP, Ca and P were determined using the Idexx Catalyst One biochemical analyzer (Idexx Laboratories, USA) and sets of biochemical reagents produced by Idexx Laboratories (Westbrook, USA).

### Bones weight, femur and tibia osteometric measurements

Femur and tibia weights were determined using the Adventurer Pro AV513CM electronic balance with an accuracy of 0.001 g (Ohaus Europe GmbH, Nanikon, Switzerland). Based on measurements of the BW of the chickens and the weight of the bones, the relative weight of the bones (**RBW**) was calculated. The femur and tibia were then measured using a STALCO s-11115 electronic calliper with an accuracy of 0.01 mm. Bone osteometric parameters such as bone length and proximal and distal epiphysis widths were measured. From the measurements of bone mass and bone length, the Seedor Index (**SI**) was derived (Osiak-Wicha et al., 2023; Seedor et al., 1991). Osteometric parameters are quantitative macroscopic measurements of bones taken using specialized tools such as an electronic caliper, a ruler, or a measuring tape, and include, for example, measurements of bone length or width (Joshi et al., 2026).

### Bone geometric parameters

After osteometric and mechanical analyses of the bones, femur and tibia cross-sectional properties (the horizontal (M-L plane) external **HED** and internal **HID**, the vertical (A-P plane) external VED and internal VID diameters) were determined using the digital microscope (VHS-2000, Keyence Corp., Osaka, Japan) (Akkus et al., 2004; Wojciechowska-Puchałka et al., 2023). On the basis of the measurements obtained, the following geometrical parameters of the bone shaft were set:

$$\text{MRWT}\;=\frac{\frac{\left(\text{VED}\;-\;\text{VID}\right)}{\text{VID}}+\;\frac{\left(\text{HED}\;-\;\text{HID}\right)}{\text{HID}}}{2}$$

$$A=3.14\frac{\left(\left(HED\cdot VED\right)-\left(HID\cdot VID\right)\right)}{4}$$

$$\text{CI}\;=\left\{\frac{\left[\left(\frac{\left(\text{HED}-\text{HID}\right)}{\text{HED}}\right)+\left(\frac{\text{VED}-\text{VID}}{\text{VED}}\right)\right]}{2}\right\}100$$

where **MRWT** is mean relative wall thickness, **A** is cortical cross-sectional area, **CI** is cortical index, HED is horizontal external diameter, HID is horizontal internal diameter, VED is vertical external diameter, VID is vertical internal diameter (Brzóska et al., 2005; Osiak-Wicha et al., 2023).

Moreover, the parameters (which do not specify the geometrical parameters of the bone) necessary to set the strength and material properties of the bone were determined:

$$I_{x}=\frac{\pi }{64}\left(HED\cdot VED^{3}-HID\cdot \text{VE}D^{3}\right)$$

$$R_{g}=\sqrt{\frac{I_{x}}{A}}$$

where **I_x_** is mean moment of inertia, R_g_ is radius of gyration, HED is horizontal external diameter, HID is horizontal internal diameter, VED is vertical external diameter, VID is vertical internal diameter, A is cortical cross-sectional area (Muszyński et al., 2017). Geometric parameters are a set of measures describing the size, shape, and distribution of bone tissue in a cross-section, which determine the mechanical strength of bone (Augat et al., 1996; Charuta et al., 2013).

### Bone mechanical parameters

The tibia and femur were tested in a three-point bending test using a Zwick Z010 testing machine (Zwick GmbH & Company KG, Ulm, Germany) connected to a recording computer with TestXpert II 3.1 software (Zwick GmbH & Company KG, Ulm, Germany). Measurements were made at a load rate of 3 mm/min, with support distance equal to 12 mm and 8 mm, for tibia and femur, respectively. The bones were loaded in the anterior posterior (A-P) plane until fracture. The bone strength parameters were obtained from the load-displacement curves using Origin software (ver. 2022, OriginLab, Northampton, MA, USA). The yield load **F_y_**, ultimate load F_max_, elastic deformation **x_y_** and deformation at fracture point **x_max_** were determined. Furthermore, the following strength properties were calculated:

$$W_{y}=\frac{1}{2}\cdot F_{y}\cdot x_{y}$$

$$W_{\text{max}}=\frac{1}{2}F_{\text{max}}\cdot x_{\text{max}}$$

$$S=\frac{F_{y}}{x_{y}}$$

$$u=\frac{3\cdot W_{max}\cdot VED^{2}}{4\cdot I_{x}\cdot L\;}$$

where **W_y_** is elastic energy, **F_y_** is yield load, **x_y_** is elastic deformation, W_max_ is work to fracture, F_max_ is ultimate load, **x_max_** is deformation at fracture point, **S** is stiffness, **u** is toughness modulus, VED is vertical external diameter, I_x_ is mean moment of inertia, **L** - support distance width in bending test (Brzóska et al., 2005; Schriefer et al., 2005).

### Bone material parameters

Based on the geometric and mechanical parameters of the bone described above, the material properties of the femur and tibia were determined:

$$E=\frac{S\cdot L^{3}}{48\cdot I_{x}}$$

$$M=\frac{F_{y}\cdot L\;}{4}$$

$$\varepsilon_{y}=\frac{6\cdot x_{y}\cdot VED}{L^{2}}$$

$$\varepsilon_{\text{max}}=\frac{6\cdot x_{\text{max}}\cdot VED}{L^{2}}$$

$$\sigma_{y}=\frac{x_{y}\cdot VED\cdot L}{8\cdot I_{x}}$$

$$\sigma \text{max}=\frac{F_{\text{max}}\cdot VED\cdot L}{8\cdot I_{x}}$$

where **E** is Young modulus, S is stiffness, I_x_ is mean moment of inertia, L - support distance width in bending test, **M** is bending moment, F_y_ is yield load, F_max_ is ultimate load, ε_y_ is yield strain, x_y_ is elastic deformation, VED is vertical external diameter, ε_max_ is ultimate strain, x_max_ is deformation at fracture point, **σ_y_** is yield stress, **σ_max_** is ultimate stress. Significantly, these indicators remain independent of bone size or strength testing conditions (Muszyński et al., 2017; Osiak-Wicha et al., 2023).

### Identification of selected bone minerals

Determination of Ca, P, potassium (**K**), sodium (**Na**) and magnesium (**Mg**) was conducted after strength testing. Femur and tibia bone fragments were dried at 60°C, weighed out about 350 mg of analytical material and added 3.5 ml of HNO_3_ (Merck, St. Louis, MO, USA) and 0.5 ml of H_2_O_2_ (Merck, St. Louis, MO, USA) in Teflon vessels. After that, microwave digestion of the samples was carried out using a Multiwave 5000 microwave system (Anton Paar, Graz, Austria) at 190°C for 30 minutes. After mineralization, the samples were quantitatively filtered into 25 ml volumetric flasks. In such prepared solutions, containing pooled femur and tibia fragments, the content of the selected elements was determined using ICP-OES spectrometer Optima7300 DV instrument (Perkin Elmer, Boston, MA, USA). The quality of determinations was verified using the recovery method, with results ranging from 96% to 102%.

### Statistical analysis

Data were analyzed using one-way analysis of variance (**ANOVA**) with Dunnett's post-hoc test to compare the control group with the several experimental groups. Normality of the data was assessed using the Shapiro-Wilk test, while homogeneity of variance was evaluated using the Brown-Forsythe test. Log transformations were applied as needed to maintain homogeneity of variance and normal distribution. Data which did not meet the mentioned requirements were compared using the non-parametric Kruskal-Wallis test with Dunn’s multiple comparisons test. Results are presented as means ± standard error (**SE**). Significance was declared at P < 0.05. All of the statistical results were obtained from Statistica 13.3 (TIBCO Software Inc., Palo Alto, USA).

## Results

### Hatchability

The hatchability rate observed in the experiment was 74.0% in the 0-ARC group, 67.7% in the L-ACR group, 66.7% in the M-ACR group, and 49.5% in the H-ACR group. However, in the groups treated with high doses of ACR (2 and 4 mg/egg), hatchability was not reported because the embryos died.

### Body weight

Analysis of the body weight results of one-day-old chicks showed no significant differences between the control group and the experimental groups (Fig. 1).

**Fig. 1:**
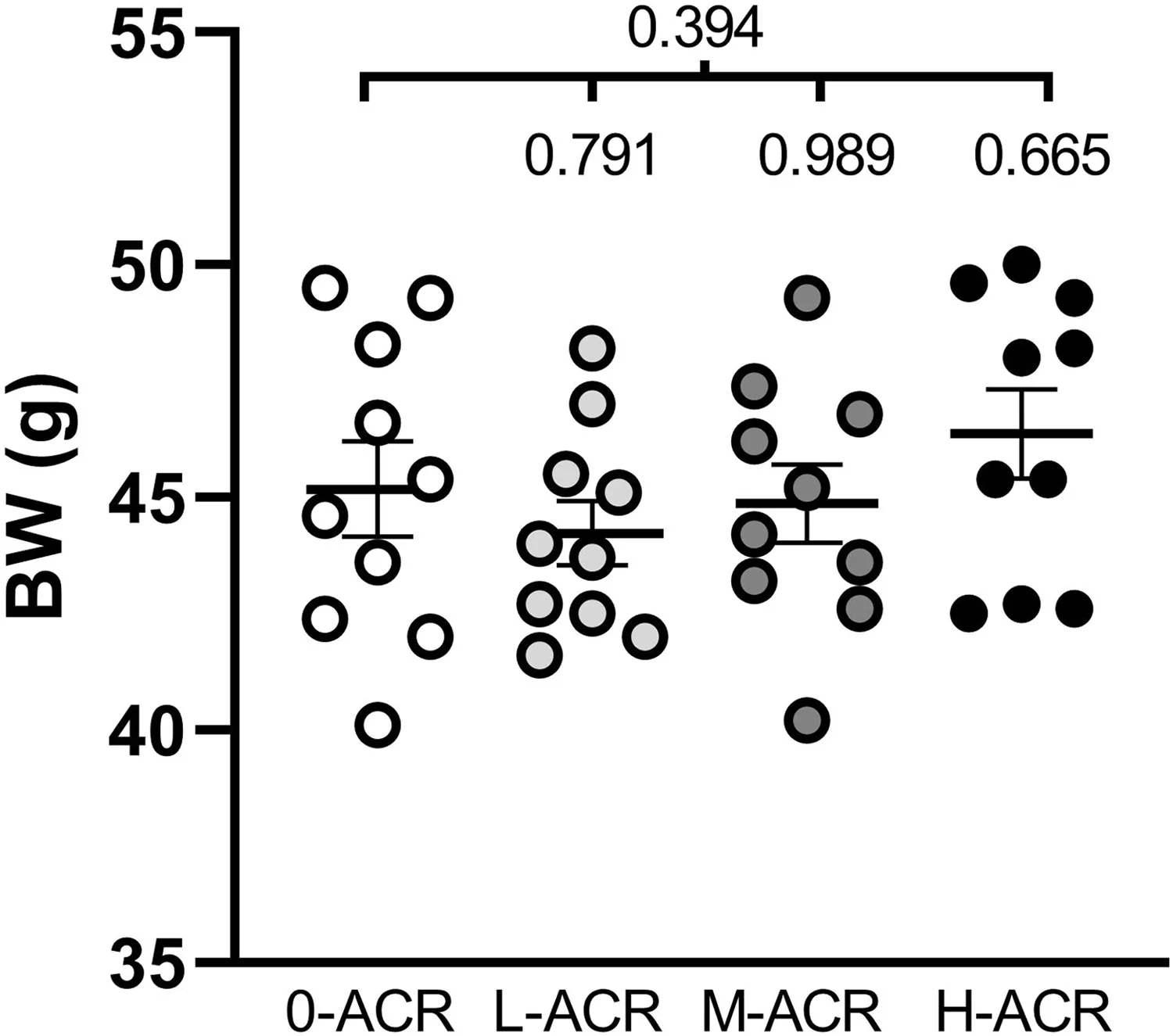
Body weight (BW) of one-day-old chicks in the control group (0-ACR) and experimental groups (L-ACR, M-ACR, H-ACR). Data are presented as mean (line) ± SE (whiskers). A p-value was attributed above plots.

### Calcium, phosphorus and alkaline phosphatase levels

There were no significant differences in the levels of Ca, P, ALP in the blood plasma of one-day-old individuals exposed *in ovo* to 0.9% saline solution and various doses of ACR (Table 1).

**Table 1:** Basal bone turnover markers in the blood plasma of one-day-old chicks.

Parameter	Groups				P value
	0-ACR	L-ACR	M-ACR	H-ACR	
Ca (mmol/L)	2.34 ± 0.02	2.45 ± 0.08	2.32 ± 0.03	2.28 ± 0.03	P = 0.170^b^
P (mmol/L)	1.40 ± 0.09	1.63 ± 0.12	1.56 ± 0.08	1.60 ± 0.07	P = 0.305^a^
Ca/P ratio (-)	1.70 ± 0.11	1.53 ± 0.10	1.51 ± 0.09	1.44 ± 0.08	P = 0.288^a^
ALP (U/L)	506.00 ± 43.22	602.83 ± 147.08	511.50 ± 44.13	479.67 ± 31.01	P = 0.986^b^

Data given are mean ± SE.

0-ACR - the control group received 0.9% saline solution; L-ACR - the experimental group received ACR at a dose of 0.25 mg/egg; M-ACR - the experimental group received ACR at a dose of 0.5 mg/egg; H-ACR - the experimental group received ACR at a dose of 1.0 mg/egg.

Ca = calcium; P = phosphorus; ALP = alkaline phosphatase.

a Results for a one-way analysis of variance (ANOVA) with Dunnett's post-hoc test.

b Results for the Kruskal-Wallis test with Dunn’s multiple comparisons test.

### Bone weight, osteometric and geometric parameters

The administration of ACR to chicken embryos at a dose of 0.5 mg per egg increased the VED (P = 0.025, Table 2) and R_g_ (P = 0.034, Table 2) values of the tibia and the VID (P = 0.014, Table 3) of the femur compared to the control group treated with 0.9% saline solution. Significantly, such effects were not observed in groups receiving 0.25 mg and 1 mg ACR per egg. However, intoxication with this substance at various doses did not affect the other osteometric and geometric parameters of the tibia (Table 2) and femur (Table 3).

**Table 2:** Osteometric and geometric parameters of the tibia bone among one-day-old chicks.

Parameter	Groups				P value
	0-ACR	L-ACR	M-ACR	H-ACR	
Osteometric properties					
Bone weight (g)	0.19 ± 0.01	0.21 ± 0.01	0.20 ± 0.01	0.19 ± 0.01	P = 0.390^a^
RBW (%)	0.42 ± 0.02	0.48 ± 0.02	0.45 ± 0.02	0.42 ± 0.02	P = 0.115^a^
Bone length (mm)	30.06 ± 0.28	29.99 ± 0.32	29.98 ± 0.33	29.73 ± 0.30	P = 0.881^a^
Proximal epiphysis width (mm)	6.50 ± 0.15	6.40 ± 0.15	6.40 ± 0.12	6.40 ± 0.15	P = 0.946^b^
Distal epiphysis width (mm)	5.60 ± 0.15	5.45 ± 0.12	5.30 ± 0.13	5.45 ± 0.14	P = 0.470^b^
SI (mg/mm)	6.34 ± 0.36	7.12 ± 0.27	6.69 ± 0.26	6.47 ± 0.29	P = 0.290^a^
Geometric properties					
HED (mm)	2.04 ± 0.05	2.15 ± 0.04	2.07 ± 0.07	1.99 ± 0.09	P = 0.397^a^
HID (mm)	1.42 ± 0.06	1.47 ± 0.04	1.44 ± 0.04	1.35 ± 0.07	P = 0.455^a^
VED (mm)	1.62 ± 0.04	1.77 ± 0.04	1.83 ± 0.07*	1.78 ± 0.06	P = 0.049^a^
VID (mm)	1.04 ± 0.05	1.11 ± 0.04	1.17 ± 0.06	1.10 ± 0.06	P = 0.398^a^
MRWT (-)	0.51 ± 0.05	0.53 ± 0.04	0.51 ± 0.03	0.58 ± 0.06	P = 0.807^a^
A (mm^2^)	1.43 ± 0.10	1.69 ± 0.09	1.65 ± 0.13	1.63 ± 0.12	P = 0.158^b^
CI (%)	33.06 ± 2.20	34.25 ± 1.61	33.11 ± 1.41	35.45 ± 2.32	P = 0.792^a^
I_x_ (mm^4^)	0.35 ± 0.04	0.49 ± 0.04	0.53 ± 0.07	0.49 ± 0.06	P = 0.126^a^
R_g_ (mm)	0.49 ± 0.01	0.53 ± 0.01	0.55 ± 0.02*	0.53 ± 0.02	P = 0.043^a^

Data given are mean ± SE.

0-ACR - the control group received 0.9% saline solution; L-ACR - the experimental group received ACR at a dose of 0.25 mg/egg; M-ACR - the experimental group received ACR at a dose of 0.5 mg/egg; H-ACR - the experimental group received ACR at a dose of 1.0 mg/egg.

RBW = relative bone weight; SI = Seedor Index; HED = horizontal external diameter; HID = horizontal internal diameter; VED = vertical external diameter; VID = vertical internal diameter; MRWT = mean relative wall thickness; A = cortical cross-sectional area; CI = cortical index; I_x_ = moment of inertia; R_g_ = radius of gyration.

Significant differences compared to the control values are marked with asterisks.

* P < 0.05.

a Results for a one-way analysis of variance (ANOVA) with Dunnett's post-hoc test.

b Results for the Kruskal-Wallis test with Dunn’s multiple comparisons test.

**Table 3:** Osteometric and geometric parameters of the femur bone among one-day-old chicks.

Parameter	Groups				P value
	0-ACR	L-ACR	M-ACR	H-ACR	
Osteometric properties					
Bone weight (g)	0.10 ± 0.01	0.10 ± 0.01	0.12 ± 0.01	0.11 ± 0.01	P = 0.537^b^
RBW (%)	0.23 ± 0.01	0.22 ± 0.03	0.26 ± 0.01	0.23 ± 0.02	P = 0.541^b^
Bone length (mm)	21.93 ± 0.26	21.32 ± 0.32	21.61 ± 0.24	21.35 ± 0.29	P = 0.388^a^
Proximal epiphysis width (mm)	5.00 ± 0.07	5.00 ± 0.00	5.00 ± 0.00	4.90 ± 0.10	P = 0.789^b^
Distal epiphysis width (mm)	5.25 ± 0.11	5.25 ± 0.13	5.10 ± 0.07	5.20 ± 0.11	P = 0.788^b^
SI (mg/mm)	4.70 ± 0.32	4.56 ± 0.55	5.32 ± 0.25	4.99 ± 0.32	P = 0.496^a^
Geometric properties					
HED (mm)	1.93 ± 0.07	1.97 ± 0.06	2.00 ± 0.05	2.00 ± 0.06	P = 0.841^a^
HID (mm)	1.40 ± 0.05	1.38 ± 0.04	1.37 ± 0.05	1.35 ± 0.04	P = 0.896^a^
VED (mm)	1.58 ± 0.04	1.65 ± 0.05	1.71 ± 0.05	1.60 ± 0.03	P = 0.219^a^
VID (mm)	0.96 ± 0.07	1.10 ± 0.04	1.17 ± 0.04*	1.03 ± 0.03	P = 0.031^a^
MRWT (-)	0.55 ± 0.07	0.47 ± 0.03	0.46 ± 0.01	0.52 ± 0.03	P = 0.474^b^
A (mm^2^)	1.34 ± 0.11	1.37 ± 0.10	1.41 ± 0.05	1.40 ± 0.05	P = 0.900^a^
CI (%)	33.35 ± 2.65	31.62 ± 1.33	31.43 ± 0.68	33.72 ± 1.24	P = 0.797^a^
I_x_ (mm^4^)	0.31 ± 0.03	0.35 ± 0.04	0.39 ± 0.04	0.33± 0.02	P = 0.425^a^
R_g_ (mm)	0.48 ± 0.02	0.50 ± 0.01	0.52 ± 0.02	0.48 ± 0.01	P = 0.364^a^

Data given are mean ± SE.

0-ACR - the control group received 0.9% saline solution; L-ACR - the experimental group received ACR at a dose of 0.25 mg/egg; M-ACR - the experimental group received ACR at a dose of 0.5 mg/egg; H-ACR - the experimental group received ACR at a dose of 1.0 mg/egg.

RBW = relative bone weight; SI = Seedor Index; HED = horizontal external diameter; HID = horizontal internal diameter; VED = vertical external diameter; VID = vertical internal diameter; MRWT = mean relative wall thickness; A = cortical cross-sectional area; CI = cortical index; I_x_ = moment of inertia; R_g_ = radius of gyration.

Significant differences compared to the control values are marked with asterisks.

*P < 0.05.

a Results for a one-way analysis of variance (ANOVA) with Dunnett's post-hoc test.

b Results for the Kruskal-Wallis test with Dunn’s multiple comparisons test.

### Bone mechanical parameters

A significant increase in F_max_ (P = 0.022, Table 4) and W_max_ (P = 0.036, Table 4) was observed in the tibia in the L-ACR group compared to the 0-ACR group. Such relationships were not observed in individuals from the M-ACR and H-ACR groups (Table 4). In the case of mechanical parameters of the tibia, such as F_y_, W_y_, S, and u, the differences between the individual experimental groups and the control group were not statistically significant (Table 4). Importantly, ACR poisoning did not have a significant effect on all mechanical parameters of the femur (Table 4).

**Table 4:** Mechanical parameters of the tibia and femur bone among one-day-old chicks.

Parameter	Groups				P value
	0-ACR	L-ACR	M-ACR	H-ACR	
Tibia bone					
F_y_ (N)	5.24 ± 0.14	6.06 ± 0.29	5.82 ± 0.35	5.63 ± 0.18	P = 0.109^b^
F_max_ (N)	6.86 ± 0.24	8.21 ± 0.39*	7.96 ± 0.42	7.54 ± 0.28	P = 0.043^a^
W_y_ (mJ)	1.08 ± 0.06	1.40 ± 0.12	1.30 ± 0.12	1.33 ± 0.09	P = 0.148^a^
W_max_ (mJ)	3.99 ± 0.26	5.11 ± 0.40*	4.80 ± 0.39	4.60 ± 0.21	P = 0.034^a^
S (N/mm)	12.73 ± 0.43	13.24 ± 0.80	13.33 ± 0.95	12.23 ± 0.70	P = 0.706^a^
u (mJ/mm^3^)	1.96 ± 0.17	2.11 ± 0.16	2.00 ± 0.12	2.13 ± 0.23	P = 0.879^a^
Femur bone					
F_y_ (N)	6.76 ± 0.26	6.71 ± 0.34	6.67 ± 0.37	6.79 ± 0.46	P = 0.996^a^
F_max_ (N)	8.64 ± 0.52	9.61 ± 0.51	8.62 ± 0.55	8.77 ± 0.53	P = 0.514^a^
W_y_ (mJ)	1.56 ± 0.10	1.30 ± 0.10	1.77 ± 0.24	1.70 ± 0.20	P = 0.263^a^
W_max_ (mJ)	4.32 ± 0.28	5.16 ± 0.33	4.71 ± 0.38	4.55 ± 0.41	P = 0.396^a^
S (N/mm)	15.51 ± 1.36	19.10 ± 1.77	14.02 ± 1.38	16.24 ± 1.34	P = 0.118^a^
u (mJ/mm^3^)	3.12 ± 0.27	3.70 ± 0.30	3.18 ± 0.26	3.17 ± 0.32	P = 0.606^b^

Data given are mean ± SE.

0-ACR - the control group received 0.9% saline solution; L-ACR - the experimental group received ACR at a dose of 0.25 mg/egg; M-ACR - the experimental group received ACR at a dose of 0.5 mg/egg; H-ACR - the experimental group received ACR at a dose of 1.0 mg/egg.

F_y_ = yield load; F_max_ = ultimate load; W_y_ = elastic energy; W_max_ = work to fracture; S = stiffness; u = toughness modulus.

Significant differences compared to the control values are marked with asterisks.

*P < 0.05.

a Results for a one-way analysis of variance (ANOVA) with Dunnett's post-hoc test.

b Results for the Kruskal-Wallis test with Dunn’s multiple comparisons.

### Bone material parameters

Analysis of the material parameters of the tibia of one-day-old chicks showed a significant increase in ε_y_ (P = 0.002 in the case L-ACR, P = 0.003 in the case M-ACR and P < 0.001 in the case H-ACR, Table 5) and ε_max_ (P = 0.009 in the case L-ACR, P = 0.010 in the case M-ACR and P = 0.016 in the case H-ACR, Table 5) in all experimental groups compared to the control group. However, no significant changes were found in the E, M, σ_y_ and σ_max_ of the tibia of ACR-poisoned individuals (Table 5). In turn, in the case of the femur, only an increase in ε_max_ (P = 0.032, Table 5) was observed in the group of birds injected with a dose of 0.5 mg ACR per egg compared to individuals treated with a 0.9% saline solution. Such relationships were not noted in groups treated with other doses of the toxic substance (Table 5). In addition, the material parameters of the femur, such as E, M, ε_y_, σ_y_, and σ_max_, were similar in all treatment groups (0-ACR, L-ACR, M-ACR, H-ACR) and statistically did not differ significantly (Table 5).

**Table 5:** Material parameters of the tibia and femur bone among one-day-old chicks.

Parameter	Groups				P value
	0-ACR	L-ACR	M-ACR	H-ACR	
Tibia bone					
E (MPa)	1429.21 ± 145.94	1051.29 ± 108.51	996.01 ± 85.02	1149.35 ± 143.62	P = 0.176^a^
M (Nm)	15.78 ± 0.52	18.18 ± 0.95	17.50 ± 1.17	16.72 ± 0.54	P = 0.267^b^
ε_y_ (-)	2.10 ± 0.10	2.81 ± 0.13**	2.79 ± 0.13**	2.91 ± 0.17***	P < 0.001^a^
ε_max_ (-)	5.12 ± 0.21	6.47 ± 0.35**	6.45 ± 0.37*	6.38 ± 0.26*	P = 0.007^a^
σ_y_ (MPa)	38.65 ± 2.75	34.97 ± 2.91	32.66 ± 2.15	36.32 ± 5.24	P = 0.633^a^
σ_max_ (MPa)	50.17 ± 3.00	47.13 ± 3.53	44.67 ± 2.66	48.88 ± 7.32	P = 0.597^b^
Femur bone					
E (MPa)	558.01 ± 50.63	627.49 ± 81.42	412.12 ± 46.84	546.12 ± 55.82	P = 0.199^b^
M (Nm)	14.82 ± 0.61	14.32 ± 0.77	14.46 ± 0.87	14.44 ± 0.92	P = 0.974^a^
ε_y_ (-)	5.65 ± 0.51	5.00 ± 0.34	6.95 ± 0.87	6.28 ± 0.40	P = 0.133^b^
ε_max_ (-)	10.63 ± 0.55	11.93 ± 0.60	12.76 ± 0.59*	11.57 ± 0.54	P = 0.031^a^
σ_y_ (MPa)	36.43 ± 3.05	33.47 ± 3.11	30.76 ± 2.16	34.20 ± 3.04	P = 0.578^a^
σ_max_ (MPa)	46.40 ± 3.98	47.62 ± 3.91	39.82 ± 3.05	43.92 ± 3.51	P = 0.452^a^

Data given are mean ± SE.

0-ACR - the control group received 0.9% saline solution; L-ACR - the experimental group received ACR at a dose of 0.25 mg/egg; M-ACR - the experimental group received ACR at a dose of 0.5 mg/egg; H-ACR - the experimental group received ACR at a dose of 1.0 mg/egg.

E = Young modulus; M = bending moment; ε_y_ = yield strain; ε_max_ = ultimate strain; σ_y_ = yield stress; σ_max_ = ultimate stress.

Significant differences compared to the control values are marked with asterisks.

*P < 0.05; **P < 0.01; ***P < 0.001.

a Results for a one-way analysis of variance (ANOVA) with Dunnett's post-hoc test.

b Results for the Kruskal-Wallis test with Dunn’s multiple comparisons test.

### Bone mineral composition

While ACR administration had no effect on the Ca/P ratio and the K, Na, and Mg (Table 6) content in the pooled femur and tibia fragments of one-day-old chicks, a significant increase in Ca and P (P = 0.020 and P = 0.041, respectively, Table 6) content was observed, but only in the M-ACR group compared to the 0-ACR group.

**Table 6:** Mineral composition of the pooled femur and tibia bone fragments among one-day-old chicks.

Parameter	Groups				P value
	0-ACR	L-ACR	M-ACR	H-ACR	
Ca (mg/g)	63.26 ± 1.48	66.87 ± 1.45	69.02 ± 0.94*	65.47 ± 1.67	P = 0.049^a^
P (mg/g)	40.55 ± 0.99	42.01 ± 0.75	43.71 ± 0.63*	41.79 ± 1.06	P = 0.042^a^
Ca/P ratio (-)	1.56 ± 0.01	1.59 ± 0.01	1.58 ± 0.02	1.57 ± 0.01	P = 0.269^a^
K (mg/g)	7.71 ± 0.24	7.19 ± 0.17	7.10 ± 0.14	7.19 ± 0.14	P = 0.085^a^
Na (mg/g)	10.99 ± 0.22	10.45 ± 0.21	10.52 ± 0.25	10.66 ± 0.23	P = 0.373^a^
Mg (mg/g)	1.65 ± 0.04	1.74 ± 0.04	1.75 ± 0.03	1.70 ± 0.04	P = 0.264^a^

Data given are mean ± SE.

0-ACR - the control group received 0.9% saline solution; L-ACR - the experimental group received ACR at a dose of 0.25 mg/egg; M-ACR - the experimental group received ACR at a dose of 0.5 mg/egg; H-ACR - the experimental group received ACR at a dose of 1.0 mg/egg.

Ca = calcium; P = phosphorus; K = potassium; Na = sodium; Mg = magnesium.

Significant differences compared to the control values are marked with asterisks.

*P < 0.05.

a - Results for a one-way analysis of variance (ANOVA) with Dunnett's post-hoc test.

## Discussion

Already in the early stages of embryonic development, the skeleton is one of the first systems to form, performing numerous and extremely important roles. Significantly, it is the point of attachment for skeletal muscles, with which it forms the musculoskeletal system, enabling the body to move and function properly (Nakamura et al., 2019). It is widely known that intensive skeletal development in poultry occurs in the first weeks of life (Damaziak et al., 2019; Sanchez-Rodriguez et al., 2019). During growth and maturation, the rate of the increase gradually decreases until peak bone mass is reached, accompanied by modifications in bone dimensions and geometric structure. The process of bone remodeling is influenced by numerous factors, such as genetics, hormone levels, nutrition, age, gender, and exposure to toxic substances (Rath et al., 2000; Tykałowski et al., 2010). Among substances with mutagenic, teratogenic, and neurotoxic effects there is ACR, which is commonly found in food products as well as in animal feed, especially for poultry, which may consequently pose a risk of its transfer to chicken eggs. In recent years, there have been few studies confirming the negative impact of ACR on bone development in mammals (Martiniakova et al., 2020; Sarocka et al., 2019). But experiments on how this toxic compound affects the skeleton of offspring when administered prenatally are rare (Tomaszewska et al., 2020; Zhu et al., 2021). Importantly, such studies on poultry, especially on the *in ovo* model, do not exist. Therefore, this study focuses on assessing the effect of ACR *in ovo* administration on the skeletal system of hatched chicks. Due to the lack of detailed studies of the skeletal system in such a model, we focused our research on the analysis of morphometric, geometric, and mechanical properties of the femur and tibia, as well as the analysis of basic markers of bone turnover and bone mineral composition. We hypothesized that *in ovo* ACR exposure would dose-dependently disrupt endochondral ossification and cortical modeling and weaken long bones at hatch, with larger effects in the tibia. The results do not support this directional hypothesis. Body weight and circulating Ca, P, Ca:P, and ALP were unchanged, and the significant skeletal differences were limited to particular endpoints and exposure groups. Most importantly, the pattern was not monotonic: several differences occurred in the L-ACR or M-ACR group, whereas the H-ACR group often did not differ from the control. The findings should therefore be interpreted as selective responses detected at hatch, not as evidence of either progressive toxicity or dose-dependent stimulation.

In incubation studies, hatchability is one of the most important indicators examined. It is important to note that hatching results depend on the biological quality of the eggs, which is determined by genetic factors, the age of the flock, nutrition, and the housing conditions of the breeding birds (Biesek et al., 2023; Biesiada-Drzazga, 2020). Thus, the incubation process itself has a significant impact on poultry production, as the birds’ production performance is directly linked to the quality of one-day-old chicks and affects the profitability of the operation (Mesquita et al., 2021). In this experiment, the hatchability rate was 74.0% in the control group, while in the experimental groups it was 67.7% at a dose of 0.25 mg ACR per egg, 66.7% at a dose of 0.5 mg ACR per egg, and 49.5% at a dose of 1 mg ACR per egg, while the groups treated with doses of 2 and 4 mg ACR per egg showed complete mortality (Wojciechowska-Puchałka et al., 2026). A higher mortality rate following ACR poisoning may indicate certain developmental abnormalities. As indicated by Becit-Kizilkaya et al. (2024), ACR delays neural tube closure and affects BRE gene expression in chicken embryos and affects the availability of nutrients in the egg, thereby limiting embryonic development and jeopardizing survival during hatching. This clearly indicates the need to investigate the effects of embryonic exposure to ACR on the skeletal system.

One of the important indicators of the proper condition of the organism is the measurement of body weight. What is very important is that the body weight of birds depends partly on genetic characteristics, partly on the environment, and sometimes also on genotype-environment interactions. In addition, the body weight of broiler chickens is an important economic factor in the poultry production, which is the fastest growing sector of animal production (Adaszyńska-Skwirzyńska et al., 2025). Broiler breeding aims to achieve rapid growth in birds, and the quality of nutrition largely determines the rate of growth and final body weight of the animals (Petracci et al., 2015; Tallentire et al., 2016). Therefore, toxic substances can reduce the quality of food consumed and thus affect the condition of the body. Some studies show that ACR intoxication reduces the body weight of animals (Donmez et al., 2020; Hamdy et al., 2012; Karimani et al., 2021). However, it is worth noting that these experiments were conducted on mammals in the later stages of postnatal development. Works on embryo models or one-day-old birds or mammals are rare. Thus, Zhu et al. (2021) conducted such studies on female Sprague Dawley rats, which were administered ACR orally at doses of 5, 10, and 15 mg/kg bw/day for a period of 3 weeks after giving birth. In addition, they conducted analyses to trace the effects of ACR exposure at various stages of pregnancy, where females were administered oral doses at different levels (0.05, 1, 5, and 10 mg/kg bw/day), starting 10 days before fertilization until the day of embryo collection. And so, the authors observed that exposure of female rats to this toxic compound during lactation impaired the growth of newborns, while exposure of pregnant female rats to ACR during pregnancy led to a reduction in embryo weight on GD19, but only in the group with high doses of exposure to this substance. A somewhat different result was obtained by Swamy et al. (2011) in studies conducted on Bobcock chicken embryos treated with various doses of ACR (0.2 mg, 0.4 mg and 0.6 mg). They demonstrated a significant increase in body weight in 11-day-old embryos exposed to a dose of 0.4 mg of the toxic substance compared to those in the control group, while the differences between the other experimental groups and the control group were not statistically significant. Similar relationships were observed in this study, where the body weight of one-day-old chicks was similar in the group treated with 0.9% saline and in the groups treated with different doses of ACR. Also, research conducted by Tomaszewska et al. (2020) on female Himalayan guinea pigs receiving an aqueous solution of ACR at a dose of 3.0 mg/kg bw/day during the last 35 days of pregnancy and their offspring indicates that the administration of this substance during pregnancy has no effect on the body weight of newborns (both female and male). Similar observations regarding the lack of influence of ACR on this parameter were also made by Kienzle et al. (2005), Yerlikaya and Yener (2013), Martiniakova et al. (2020), Pazarcı et al. (2022), but it should be emphasized that in these studies, the toxic substance was introduced during the postnatal life. Therefore, the differences in animal body weight after exposure to ACR, demonstrated in many studies, are most likely due to the use of different species, doses of the compound, exposure time or various timing of its administration (prenatal/postnatal). In our study, the stable hatch weight across groups indicates that any bone effects occurred without generalized growth restriction, which narrows the interpretation to bone-intrinsic rather than systemic failure to thrive, related not only to the skeletal system. However, considering that nutritional and environmental factors influence chicken growth, feed intake, health, and carcass quality, and that the initial body weight of chicks determines the final performance of birds and thus their body weight (Adaszyńska-Skwirzyńska et al., 2025; Mendes et al., 2011), it is reasonable to continue with an experimental model similar to the one used in this study. But at that point, it's worth extending the chicks' lives to get more data on potential production impacts.

Important information about bone tissue metabolism is provided by certain biochemical indicators. It can be measured in plasma or blood serum and urine with analysis of its concentration providing information about the rate and nature of changes occurring in bone tissue (Cavalier et al., 2016). However, it should be remembered that many such indicators also originate from non-skeletal tissues. One such parameter is ALP, which is present in blood serum and includes enzymes originating not only from bones, but also from the liver, kidneys, and intestines. It is considered to be a specific marker of bone formation, bearing in mind that 50-60% of ALP comes from osteoblast production, about 30% from the intestines, and 10-20% from the liver (Charles et al., 1992; Masrour Roudsari and Mahjoub, 2012). Importantly, its bone isoform dominates during growth and maturation (Poynard and Imbert-Bismut, 2012). The available literature contains studies assessing the effects of ACR on ALP levels, but these mainly concern mature individuals (Raju et al., 2015; Tomaszewska et al., 2022). There is a lack of works analyzing the impact of this substance during the prenatal period (after administration of ACR to mothers) in mammals or after *in ovo* administration in birds. Our own research has shown that the administration of ACR to chicken embryos has no effect on the level of ALP in the blood of one-day-old chicks, which is consistent with earlier observations by Raju et al. (2016), Pazarcı et al. (2022) and Sarocka et al. (2019) on older mammalian models. However, Donmez et al. (2020), Karimani et al. (2021) and Rivadeneyra-Domínguez et al. (2018) demonstrated in their experiments on adult individuals an increase in blood ALP levels in individuals treated with ACR compared to those in the control group. Such an increase in the activity of this biochemical indicator among animals exposed to ACR may indicate increased bone turnover in their organisms and possibly indicate bone formation as a result of increased resorption (Romagnoli et al., 1998). In contrast, an experiment conducted by Rawi et al. (2012), which used younger male and female rats (21 days old), shows different results. Thus, animals that received ACR at a dose of 15 mg/kg/bw for 28 days demonstrated a decrease in blood ALP levels compared to rats that were administered distilled water. This may indicate a negative impact of this toxic substance on bone metabolism, because the physiological increase in ALP activity occurs during skeletal growth, and a decrease in its level should only occur after growth (Jwa et al., 2013; Tobiume et al., 1997). On the other hand, the studies cited above concerned the entire fraction of this enzyme, which does not fully reflect the processes occurring in bone tissue. Additionally, the mechanisms regulating ALP activity are not fully understood, and it is generally believed that the BMP/RUNX2 and Wnt metabolic pathways, as well as PTH, vitamin 1,25(OH)_2_D, and retinoids, are involved in these mechanisms (Golub and Boesze-Battaglia, 2007). Therefore, it would be reasonable to initiate further experiments based on appropriate isoenzymes with greater diagnostic value, as well as other hormones and bone turnover markers involved in bone metabolism.

A more complete insight into the condition of the skeletal system is also provided by measurements of Ca and P levels, which are the basic builders of bone tissue and are responsible for maintaining proper mineral balance throughout the organism (Penido and Alon, 2012; Voulgaridou et al., 2023). Many studies conducted on mammals show that ACR intoxication does not affect the Ca level in the blood of animals (but in juvenile and adult rats) (Hammad et al., 2013; Takami et al., 2012; Yener et al., 2016). This is consistent with the results obtained in this study, where we also did not observe significant changes in Ca concentration in the blood plasma of hatched chicks treated during embryonic development with various doses of ACR and 0.9% saline solution. Interestingly, only Sarocka et al. (2019) demonstrated an increase in the level of this element in the blood plasma of mice receiving ACR compared to individuals not supplemented with this toxic substance. This may indicate increased Ca release from bones, which is reflected in increased Ca levels in blood serum/plasma. On the other hand, in the case of P, numerous experiments also indicate that ACR has no effect on the level of this macro element in the blood of rodents in which the experimental procedures were carried out in later stages of ontogenetic development (Martiniakova et al., 2020; Raju et al., 2016; Yerlikaya and Yener, 2013) . What's noteworthy is that similar relationships were observed in this study in one-day-old chicks after *in ovo* administration of ACR. Only the observations of Pazarcı et al. (2022) indicate a decrease in blood P levels in adult Albino rats treated ACR compared to individuals from control group I. However, Hammad et al. (2013) reported a significant increase in serum P concentrations in young male Wistar rats fed with feed containing this toxic substance at doses of 10 mg/kg, 30 mg/kg, and 90 mg/kg for 6 weeks compared to animals receiving a basic diet. In addition, the authors observed a decrease in the level of this element during the 4-week recovery period, but only in individuals poisoned with a dose of 60 mg/kg ACR compared to rats from the control group. Thus, measuring Ca and P concentrations in serum/blood plasma has some diagnostic value, but also limits the detection of bone tissue metabolism disorders to some extent. It should be noted that the metabolism of these minerals depends on many factors (diet, PTH or hormones) and is related not only to the skeletal system, but also to the urinary and digestive systems. What is important is that the stable concentrations of Ca, P, the Ca:P ratio, and total ALP in plasma in our study provide no evidence of a systemic mineral imbalance at the time of hatching. In poultry embryos, mineral transfer from the eggshell and yolk intensifies late in incubation, while the skeleton at hatch remains rapidly developing (Torres and Korver, 2018). Circulating mineral concentrations are also homeostatically regulated and total ALP is not bone-specific. Localized or transient changes in bone formation may therefore have occurred without being reflected in the circulating markers measured at hatch.

Importantly, the femur and tibia are major long bones in the bird skeleton that carry primary physiological loads and perform locomotor functions (Sanchez-Rodriguez et al., 2019; Wojciechowska-Puchałka et al., 2023). It is known that ACR is a toxic substance that affects the neural tube, reduces physical activity, and may affect bone tissue metabolism during the embryonic period and after hatching/birth (Becit-Kizilkaya et al., 2024; El-Bakry et al., 2013; Seale et al., 2012). Despite such clear connections, there is a lack of research in the available literature on both prenatal and postnatal exposure to this toxic substance and its impact on the structure and mechanics of the skeletal system among animals. Martiniakova et al. (2020) compared the weight and length of the femur of 14-week-old Swiss mice treated for two weeks with ACR at a dose of 20 mg/kg bw/day and those not receiving the toxic substance, and found no significant differences between the groups. However, only Tomaszewska et al. (2020) conducted a study on a guinea pig model to investigate maternal nutritional programming in the context of the skeletal system. Thus, the authors demonstrated that ACR administration had a negative effect on femur length, cross-sectional area and cross-sectional moment of inertia of the femur. However, they did not observe its impact on bone weight, Seedor index, mean relative wall thickness and cortical index of the femur in newborns. In turn, our study revealed certain changes in the geometry of the femur and tibia of one-day-old chicks. The geometric findings were modest and confined to the intermediate exposure group: tibial VED and R_g_ and femoral VID were higher in M-ACR than in controls, while bone length, weight, A, CI, and I_x_ did not differ. At hatch, avian cortical bone contains abundant newly deposited, incompletely mineralized tissue and substantial porosity; its geometry and mineral organization change rapidly during early posthatch growth (Rawlinson et al., 2009; Sanchez-Rodriguez et al., 2019). Within this developmental context, isolated diameter differences cannot be equated with improved bone quality or with compensatory cortical modeling. Histomorphometry could provide complementary information on tissue-level developmental variation, although it would not directly explain the differences in whole-bone geometry observed here (Sanchez-Rodriguez et al., 2019). The lack of changes in the structure and geometry of the long bones of individuals exposed to other doses of the toxic substance may indicate that it does not impair these animals’ motor coordination. This is very important because it is known that ACR is neurotoxic and leads to developmental disorders, as well as increasing oxidative stress and thereby intensifying resorption, resulting in a decrease in bone mass (Zhu et al., 2021).

The direct characteristics of the supporting and protective properties of bones can be determined through experimental strength analyses conducted under controlled laboratory conditions. Such studies are similar in terms of conditions to natural physiological loads or overloads that cause fractures (Frost, 1994). Surprisingly, there is no data in the available literature showing the effect of ACR on the support properties of the skeleton. Therefore, the studies presented in this work are the first to analyze the effect of ACR administration during embryonic development on the mechanical and material parameters of the femur and tibia of hatched chicks. Mechanical responses were more evident in the tibia than in the femur. This is consistent with previous findings identifying the tibia as a particularly sensitive and representative site for evaluating skeletal development, biomechanical competence, and strength in poultry (Rath et al., 2000; Damaziak et al., 2019; Wojciechowska-Puchałka et al., 2023). Tibial F_max_ and W_max_ were higher only in L-ACR, whereas tibial yield and ultimate strains were higher in all ACR groups. Stiffness, Young's modulus, yield and ultimate stresses, and femoral whole-bone mechanical outcomes were unchanged. Thus, the exposed tibiae underwent greater deformation before yield and fracture without a corresponding loss of stress-bearing capacity in this test. This combination does not indicate weakening at hatch, but neither does it establish beneficial stimulation: whole-bone force and work depend jointly on size, cross-sectional geometry, porosity, mineralization, and matrix organization (Rath et al., 2000; Sanchez-Rodriguez et al., 2019), so the present data do not identify which component accounted for the observed changes. Furthermore, the hatched chicks showed no visible leg damage and locomotor function problems. As noted above, this response is likely tied to the high porosity and incomplete mineralization characteristic of avian cortical bone at hatch (Rawlinson et al., 2009). Furthermore, during this period of the chicks' lives, active modeling and remodeling of bone tissue occurs, which makes it difficult to interpret the results properly (Seeman and Delmas, 2006). However, both the support and load-bearing functions of the long bones of the legs of one-day-old chicks receiving ACR *in ovo* are not impaired.

The metabolic function of skeletal bones and hard tissues consists of storing minerals such as Ca, Mg and P in the form of hydroxyapatites (Ciosek et al., 2021). Ca ions in the hydroxyapatite structure can be partially replaced by Mg, K, Na, or strontium ions. Importantly, an important function of Ca, P, and Mg is to ensure the rigidity of the skeleton (Williford et al., 2008). There is no information available in the literature on the effect of ACR administration on the mineralization process and the content of minerals in the bones. Although we did not directly study the mineralization process in our work, we measured the content of certain macroelements (Ca, P, K, Na, Mg) and calculated the Ca/P molar ratio, which determines this process (Rama Rao et al., 2006; Torres and Korver, 2018). Bone Ca and P content increased together in M-ACR, while Ca:P remained unchanged. As Ca and P are major constituents of bone mineral, the Ca:P ratio provides information on their relative balance (Ciosek et al., 2021; Rama Rao et al., 2006; Torres and Korver, 2018). The parallel increase therefore reflects greater amounts of both elements in the pooled bone fragments, without evidence of a change in their relative proportions. In broilers, however, mineral content is only one of several determinants of bone strength, alongside mineral organization and structural properties (Sanchez-Rodriguez et al., 2019). Because the femur and tibia were pooled, the result cannot be assigned to a particular bone or anatomical compartment, nor does it establish increased mineral density, enhanced mineralization, or a compensatory process.

It is understood that ACR may affect bone tissue metabolism in different ways depending on the species, dose, application method, exposure time, age of the animals, and whether exposure occurred during embryonic development or later stages of life. The results obtained confirm that even small doses of ACR administered *in ovo* affect certain properties of bone tissue, which may result in the development of metabolic bone diseases during later life. As it is well known, proper growth, development, and mineralization of the skeletal system determine the physiological functions and development of the entire organism, which is important in poultry farming and may limit its economic viability. Metabolic bone diseases in legs contribute to reduced daily weight gain, lower slaughter weight, deformation of pectoral muscles, poorer feed conversion, and consequently to increased mortality in the flock. It is worth noting that the data obtained in the present study are the first in which the effect of *in ovo* ACR administration was assessed on the basis of morphometric and strength changes in the femur and tibia, as well as changes in the levels of selected macro elements comprising the bone structure among hatched chicks. As noted above, H-ACR exposure (1.0 mg/egg) reduced hatchability to 49.5% and higher doses caused complete mortality (Wojciechowska-Puchałka et al., 2026). Thus, the H-ACR exposure was biologically active, but its effects were not expressed uniformly across organs or skeletal endpoints. Within the surviving 0.25-1.0 mg/egg range, neither the skeletal variables followed a simple monotonic pattern. The absence of significant skeletal differences in many H-ACR comparisons should not be interpreted as evidence that the highest dose tested in the bone study lacked biological activity. We therefore avoid describing the skeletal pattern as stimulation, hormesis, or compensation. Demonstrating compensatory adaptation would require longitudinal recovery, histomorphometry, bone-cell markers, cortical porosity or matrix organization, and repeated posthatch measurements. As previously mentioned, avian long bones undergo pronounced changes in size, porosity, mineralization, and mechanical competence during the first weeks of life (Damaziak et al., 2019; Sanchez-Rodriguez et al., 2019). Effects detected at day 1 may be transient, may persist, or may become apparent only under the rapidly increasing mechanical demands of posthatch growth. Moreover, the lack of histology and bone-specific turnover markers prevents attribution of the observed differences to endochondral ossification, cortical formation, or resorption. Follow-up studies should therefore include serial posthatch sampling, bone histomorphometry and imaging, bone-specific biochemical markers, and locomotor outcomes.

## Author contribution

**Joanna Wojciechowska-Puchałka:** participated in the development of the research concept and methodology, assisted in the process of collecting biological samples and collecting data after hatching; prepared the research material (removal and preparation of bones) and performed an examination of the bones (their osteometric, geometric, and mechanical properties), conducted a statistical analysis, created figure and tables, and made a formal analysis and interpretation of the results obtained, reviewed the relevant literature, prepared the manuscript, provided the necessary resources and supervised the project's progress, monitored the submission of the manuscript; **Jerzy Wieczorek:** performed an analysis of selected bone minerals; reviewed the relevant literature; **Kamil Kustra:** monitored the incubation and injection of eggs, assisted in the process of collecting biological samples and collecting data after hatching; performed a biochemical blood analysis; **Magdalena Trela:** participated in the development of the research concept and methodology; monitored the incubation and injection of eggs, assisted in the process of collecting biological samples and collecting data after hatching, performed a biochemical blood analysis; **Siemowit Muszyński:** performed an examination of the bones (their osteometric, geometric, and mechanical properties); reviewed the relevant literature; **Ewa Tomaszewska:** performed an examination of the bones (their osteometric, geometric, and mechanical properties); reviewed the relevant literature; **Marcin Wojciech Lis:** participated in the development of the research concept and methodology, monitored the incubation and injection of eggs, assisted in the process of collecting biological samples and collecting data after hatching, supervised the realization of the project. All authors have evaluated, read, reviewed, and approved the final version of the manuscript and agree to take personal and public responsibility for all aspects of the work.

## Disclosures

The authors declare no conflict of interest.
